# Detection of Laryngotracheitis Virus in Poultry Flocks with Respiratory Disorders in Slovenia

**DOI:** 10.3390/v13040707

**Published:** 2021-04-19

**Authors:** Olga Zorman Rojs, Alenka Dovč, Uroš Krapež, Zoran Žlabravec, Joško Račnik, Brigita Slavec

**Affiliations:** Institute of Poultry, Birds, Small Animals and Reptiles, Veterinary Faculty, University of Ljubljana, Gerbičeva 60, 1000 Ljubljana, Slovenia; alenka.dovc@vf.uni-lj.si (A.D.); uros.krapez@vf.uni-lj.si (U.K.); zoran.zlabravec@vf.uni-lj.si (Z.Ž.); josko.racnik@vf.uni-lj.si (J.R.); brigita.slavec@vf.uni-lj.si (B.S.)

**Keywords:** chicken, infectious laryngotracheitis, partial ICP4 sequencing, mixed respiratory infections

## Abstract

Infectious laryngotracheitis (ILT) is an acute, highly contagious infectious disease of the upper respiratory tract in chickens and other poultry species that causes significant economic losses in countries worldwide. Between 2017 and 2019, seven outbreaks of mild to severe respiratory disorders with high suspicion of ILT occurred in commercial and backyard poultry flocks in Slovenia. In all submissions, infection with ILT virus (ILTV) was confirmed by PCR, which is the first report of ILT in Slovenia. Circulating ILT strains were characterized by the sequence and phylogenetic analysis of two fragments of the ICP4 gene. Four strains—three detected in non-vaccinated flocks and one in a flock vaccinated against ILT—were identical or very similar to the chicken embryo–origin live virus vaccines, and the other three were closely related to Russian, Chinese, Australian, and American field strains and to tissue culture origin vaccine strains. As in other diseases, coinfections with other respiratory pathogens in confirmed ILT cases may cause a more severe condition and prolong the course of the disease. In our study, coinfections with *Mycoplasma synoviae* (7/7 tested flocks), infectious bronchitis virus (5/5 tested flocks), *Mycoplasma gallisepticum* (4/7 tested flocks), *Ornithobacterium rhinotracheale* (3/4 tested flocks), and avian pox virus (1/2 tested flocks) were confirmed, indicating the importance of these pathogens in the occurrence of ILT infections.

## 1. Introduction

Infectious laryngotracheitis (ILT) is an acute and highly contagious respiratory disease of chickens, pheasants, peafowl, and turkeys [1,2]. The disease is caused by *Gallid herpesvirus-*1 (GaHV-1), belonging to the subfamily *Alphaherpesvirinae* in the *Herpesviridae* family. The virus has a dsDNA genome of approximately 150 kb that encodes 80 viral proteins [3].

Infection with ILT virus (ILTV) results in an economically important respiratory disease with high morbidity and variable mortality. Sources of the viral transmission include clinically ill chickens, contaminated dust, litter, beetles, drinking water and fomites. The incubation period following natural infections ranges from 6 to 14 days. The outcome of infection depends on the virulence of the strain and coinfection with other respiratory pathogens [1,2]. Two distinct clinical presentations are generally observed: a severe form characterized by dyspnea and expectoration of bloody mucus, and a milder form associated with conjunctivitis, swollen infraorbital sinuses, nasal discharge, poor egg production, and poor weight gain [4,5]. Birds that recover from GaHV-1 infection do become long-term carriers of the virus. Similar to other herpesviruses, ILTV can establish latency in the trigeminal ganglion of the central nervous system after 7 days of acute infection [6]. The virus is reactivated under the stress conditions such as onset of laying or transfer and mixing of flocks [7]. In addition, intermittent viral shedding into the trachea has been demonstrated in recovered or vaccinated chickens [8,9].

Vaccination is common practice in regions with repeated ILT outbreaks. Several types of vaccines are available, including killed, live attenuated, and recombinant vaccines [10]. Under field conditions, modified live vaccine strains propagated in chicken embryos (chicken embryo origin, CEO) or in a cell culture (tissue culture origin, TCO) are used most often. Although vaccination with live vaccines is effective, vaccine viruses can potentially revert to virulence during passage between vaccinated and unvaccinated birds. CEO vaccines have been reported to be involved in disease outbreaks in many countries worldwide. Therefore, it is common practice for live attenuated ILT vaccines to be used only in endemic areas [11].

The close antigenic and genetic relationships between field and vaccine viruses make it very difficult to distinguish between virus strains in field outbreaks [12]. PCR followed by restriction fragment length polymorphism (PCR-RFLP) has been successfully used to differentiate between field and vaccine strains [13]. However, the large amount of DNA required for this technique limits its practicality. Sequencing of specific gene fragments including the infected cell polypeptide 4 (ICP4) gene seems to be an efficient tool to characterize field isolates and differentiate them from vaccine strains. The ICP4 gene is responsible for the regulation of ILTV gene expression in the early and late stage of infection [14], and sequence analysis of the ICP4 gene is commonly used to determine the genotypic origin of the ILT strains in field infections [15,16,17].

Until 2017, ILTV had not been confirmed in Slovenia, and no vaccination against ILT was performed at commercial farms. In 2017, we started to monitor poultry flocks with respiratory signs more closely for the presence of ILTV. In this study, we report the detection of ILTV by PCR in poultry flocks with respiratory clinical signs between 2017 and 2019. The viruses detected were partially characterized by sequencing of two fragments of the ICP4 gene. In addition, the clinical signs and pathological changes in ILTV-positive flocks are also described, together with the detection of the most common respiratory pathogens, such as infectious bronchitis virus (IBV), avian pox virus (APV), *Mycoplasma gallisepticum* (MG), *Mycoplasma synoviae* (MS), and *Ornithobacterium rhinotracheale* (ORT), which could influence the outcome of the disease in ILTV-positive flocks. Samples were also tested for influenza A virus (AIV) and Newcastle disease virus (NDV) as a regular procedure in the cases of respiratory disease coupled with higher mortality in poultry flocks.

## 2. Materials and Methods

### 2.1. Affected Poultry Flocks

From August 2017 to June 2019, dead birds from seven poultry flocks with a high suspicion of ILT were submitted to the Institute of Poultry, Birds, Small Mammals, and Reptiles at the Veterinary Faculty. Four submissions were from backyard or hobby flocks and three were from commercial layer farms. In all cases, clinical and epidemiological data were collected from the submitters, and the data are presented in Table 1.

The first two cases occurred in small backyard flocks in the summer of 2017, one in hens of various ages and another in 4-week-old broilers, which were kept together with the hens without clinical signs. The following year, three outbreaks were recorded; one in a flock of 50-week-old commercial laying hens kept on a multi-age commercial farm, one in a backyard flock, and one in a hobby flock. In 2019, two commercial layer flocks were affected; one flock was imported from abroad at 17 weeks of age and kept on the multi-age farm. Mild respiratory disorders and a drop in egg production occurred at 25 weeks of age. The second case occurred in a flock of 14,000 laying hens kept on a commercial multi-age commercial farm.

Reported clinical signs included mild to severe respiratory disorders such as gasping, evident swelling of the sinuses, conjunctivitis, and nasal discharge with watery eyes. More severe disorders were described in backyard and hobby flocks. In commercial laying hens, drop in egg production was one of the most pronounced clinical signs. Low mortality was noted in all outbreaks except broilers, where 75% of the birds died within a week according to the owner (Table 1).

Routine postmortem examinations were performed in all cases. Altogether, 26 birds were pathologically examined. Then, endo- and ectoparasites observed at postmortem examination were identified by microscopic examination. For molecular investigations, oropharyngeal (including trachea) and cloacal swabs were collected. From each submission, swabs were taken from up to five birds and then pooled. Since seven outbreaks were investigated, a total of seven oropharyngeal and cloacal pooled swabs were examined by molecular analyses.

### 2.2. Molecular Analyses

Swabs were vortexed in 2 mL phosphate-buffered saline for 2 min. Total DNA and RNA were extracted from 140 µL of supernatant by the QIAamp Viral RNA Mini Kit (Qiagen, Hilden, Germany) following the manufacturer’s instructions.

Cloacal and oropharyngeal swabs were tested for AIV [18] and NDV [19] by reverse transcription (RT) real-time PCR. The oropharyngeal swabs were additionally tested for IBV [20] by RT real-time PCR, herpesvirus [21] and APV [22] by PCR, MG, and MS [23] by real-time PCR, and ORT by PCR [24]. All herpesvirus-positive samples were further tested by two sets of specific primers that amplify 688 and 635 bp fragments of the ILT ICP4 gene and are located at positions 181–204, 846–869 (ICP4-1) and 3804–3824, 4418–4440 (ICP4-2) of the ILTV ICP4 gene sequence (GenBank accession number NC_006623) [15]. The primer sequences for molecular detection of other respiratory pathogens are presented in Table 2.

The PCR volume for each set was 20 µL and contained 10 µL of DreamTag Green PCR Master Mix (2×) (Thermo Scientific, Vilnius, Lithuania), 1 µM of each PCR primer, 2 µL of isolated DNA, and deionized water up to 20 µL. The parameters for both tests were denaturation at 95 °C for 5 min; followed by 35 cycles of denaturation at 94 °C for 1 min, annealing at 62 °C for 1 min, extension at 72 °C for 1.5 min, and final extension at 70 °C for 10 min. Amplified products were separated by electrophoresis on a 1.8% agarose gel (Sigma-Aldrich, St. Louis, MO, USA) containing ethidium bromide. PCR products of 688 and 635 bp were excised and purified with a FastGene Gel/PCR extraction kit (Nippon Genetics, Düren, Germany) and sent for sequencing to Macrogen Laboratory (Macrogen Inc., Amsterdam, The Netherlands).

The nucleotide sequences obtained were first analyzed by nucleotide BLASTn [25] to identify sequences relevant for further analyses within the NCBI database. Nucleotide sequences of ICP4 gene of the following strains obtained from GenBank were used for phylogenetic analysis: WG China (JX458823.1), LJS09 China (JX458822.1), ACC78 Australia (JN804826.1), 81658 USA (JN542535.1), USDA reference USA (JN542534.1), Rus/Ck/Tatarstan/2009/1643 Russia (MF405079.1), CEO vaccine (EU104900.1), TCO vaccine (EU104908.1), CEO low passage (JN580317.1), CEO high passage (JN580316.1), TCO low passage (JN580315.1), TCO high passage (JN580314.1), TCO IVAX (JN580312.1), WangGang China (DQ995291.1), CEO vaccine strain Merial (HM230784.1), CEO vaccine strain Fort Dodge (HM230783.1), CEO vaccine strain Intervet (HM230782.1), vaccine strain A20 Australia (JN596963.1), 1874C5-USA (JN542533.1), vaccine strain SA2 Australia (JN596962.1), and live attenuated Serva vaccine (HQ630064.1) Phylogenetic trees were generated by the neighbor-joining method with the Kimura two-parameter model and 2000 bootstrap replicates by MEGA X [26].

## 3. Results

**Figure 1:**
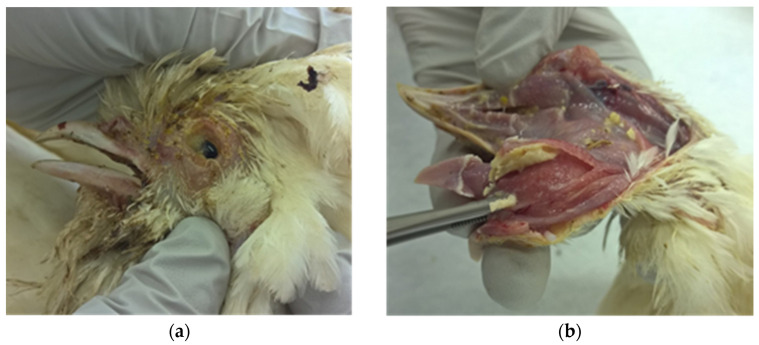
Gross pathological findings in submitted hens: (**a**) Fibrinous oculonasal discharges; (**b**) Severe fibrinous to caseous tracheitis.

The main gross pathology findings are presented in Table 3. In all birds submitted, the conjunctivae (Figure 1a) and upper respiratory tract including the sinuses, trachea, and larynx were affected, varying from mild to severe form (Figure 1b). In addition, in some cases, fibrinous airsacculitis, peritonitis, and pneumonia were found.

### 3.1. Detection of Respiratory Pathogens by PCR

All the samples were negative for AIV and NDV and positive for ILTV. Coinfection with MS was confirmed in all cases. MG, AVP, and ORT were detected in backyard flocks, and concomitant infection with IBV was found in two backyard flocks and all three submissions of commercial layer flocks (Table 3).

### 3.2. ICP4 Sequencing

**Figure 2:**
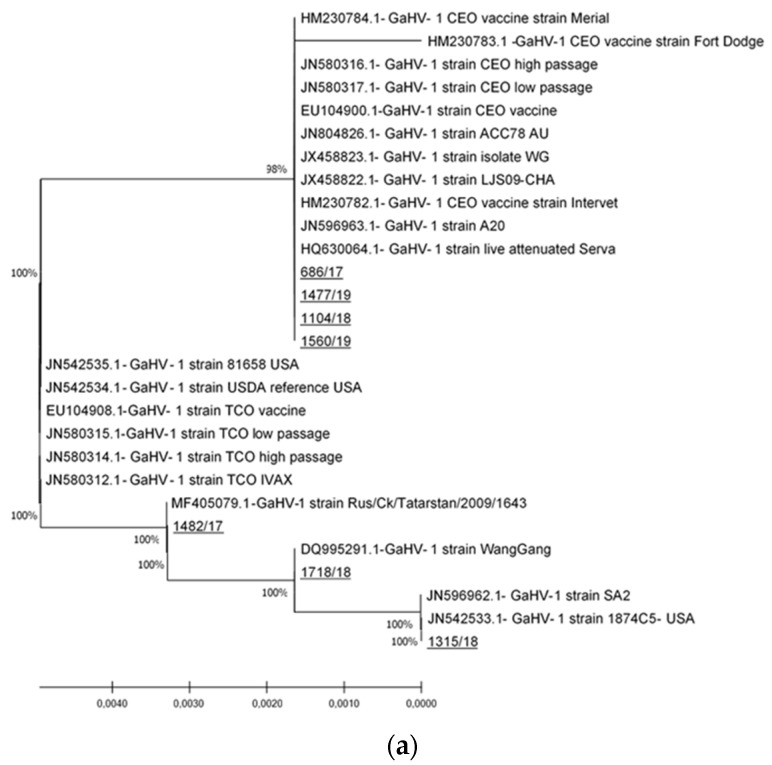

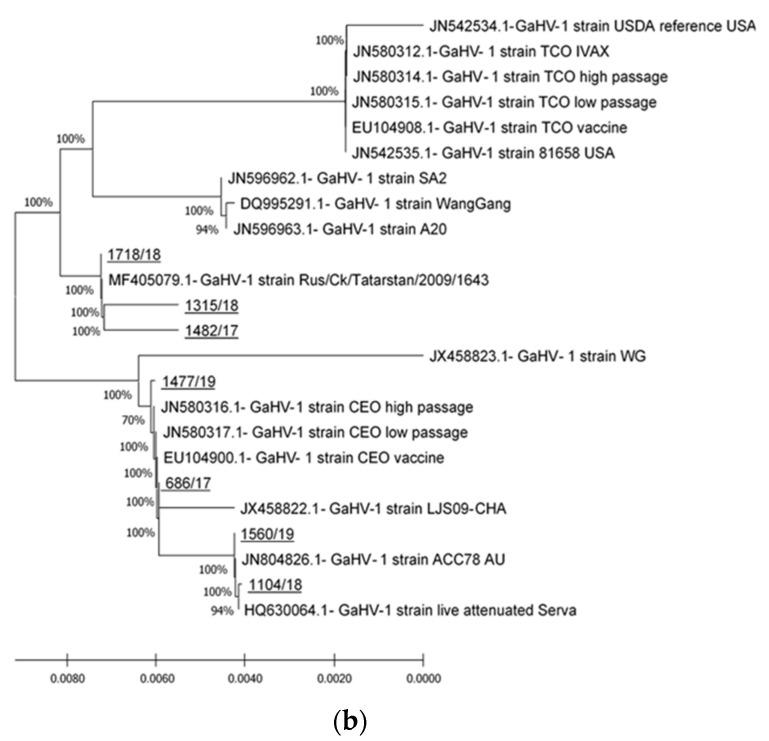
Phylogenetic relationships as calculated on partial ICP4 gene nt sequences of ILTV strains detected in Slovenia and ILTV strains derived from the GenBank database. (**a**): Phylogenetic tree on the fragment of ICP4 gene as amplified by primers ICP4-1F and ICP4-1R. (**b**): Phylogenetic tree on the fragment of the ICP4 gene as amplified by primers ICP4-2F and ICP4-2R. Both phylogenetic trees were generated by the neighbor-joining method with the Kimura-2 parameter substitution model and 2000 bootstrap replicates to assign confidence levels to branches. The scale bar indicates substitutions per site. GenBank accession numbers are given before the strain names. The nucleotide sequences obtained in this study are underlined. Phylogenetic analyses were conducted with MEGA X [25].

Phylogenetic analysis of the two ICP4 gene fragments of seven detected Slovenian ILTV strains showed that clustering of sequences of Slovenian ILTV strains does not depend on the fragment of ICP4 gene that was analyzed. The strains 686/17, 1104/18, 1477/19, and 1560/19 were grouped together with CEO vaccine strains on both gene segments, whereas the 1315/18, 1718/18, and 1560/19 strains were grouped with virulent and vaccine ILTV strains, including attenuated Australian vaccine SA2 strain on the first segment, SA2 and A20 strains on the second segment, and TCO vaccine strains on both segments (Figure 2a,b).

Sequence analyses of the first segment of the ICP4 gene (ICP4-1) in a length of 611 to 623 nucleotides (nt) showed that the sequences of four strains 686/17, 1104/18, 1477/19, and 1560/19 had 100% nt and amino acid (aa) identity, whereas the sequences from the other three strains, 1315/18, 1718/18, and 1560/19, differed from the first four. These three sequences shared 99.68 to 99.84% nt and 99.52 to 100% aa identity, respectively, and 97.27 to 97.59% nt and 97.10 to 97.58% aa identity with the aforementioned four strains due to 12 nt insertion and an additional five to three nucleotide polymorphisms in the first segment of the ICP4 gene observed in strains 1315/18, 1718/18, and 1560/19.

The analyses of sequences of the second segment of ICP4 gene (ICP4-2), 419 to 598 bp in length, revealed higher similarity between all Slovenian strains that ranged from 99.33 to 100% for nt and from 99.5 to 100% for aa identity, respectively.

## 4. Discussion

Until 2017, ILT had never been reported in Slovenia, and a non-vaccination policy against ILT had been applied in commercial flocks [11]. In a period of 2 years, infection with ILTV was confirmed in seven poultry flocks: four backyard flocks and three commercial layer flocks were affected. Reported clinical signs ranged from mild to severe respiratory disease and a decrease in egg production documented in commercial layers. It seems that younger birds were more susceptible to infection because in mixed populations of older and younger hens or broilers, the infection exhibited more severe clinical signs and caused high mortality.

Differentiation of field and vaccine ILTV strains is complicated because of their high antigenic and genetic similarity [1]. Although whole-genome sequencing of ILTV provides the most complete genetic analysis and strain differentiation [27], several recent studies have confirmed that partial sequencing and analysis of the ICP4 gene can successfully differentiate between vaccine and field ILT strains [14,15,16,28]. In our study, the identical sequences of four Slovenian ILTV strains (686/17, 1104/18, 1477/19, and 1560/19), one detected in a back-yard flock in 2017 and three detected in commercial layer flocks in 2018 and 2019, were almost identical to CEO vaccine strain sequences at the nucleotide and amino acid level and clustered together with these strains in the phylogenetic tree (Figure 2). The observed genetic cluster resembles that of Clade I as proposed by Menendez et al. This clade mainly incudes strains from the US CEO vaccines, the European CEO vaccine Serva strain, and the Australian CEO-like ACC78 virulent isolate that originated from the Serva strain [3]. Strain 1477/19 was detected in layers imported in Slovenia that were vaccinated in the rearing period with live attenuated CEO vaccine and, in this case, detection of a ILTV strain that was given as a vaccine is very likely. The other three strains, with sequences identical to the aforementioned strain detected in a vaccinated flock and almost identical to CEO vaccine strains, were detected in unvaccinated flocks. This result is not unexpected, and it is in strong agreement with previously published studies [29,30,31], suggesting that live attenuated vaccine strains have displaced wild-type viruses and may be responsible for outbreaks in non-vaccinated flocks. Since isolation and virulence determination were not performed, it is not possible to estimate the role that these strains had in the reported clinical pictures. Given that no further investigations were performed, it is unfortunately not possible to further elaborate the genetic relationship between these three strains and CEO vaccine strains that are used for vaccination in neighboring European countries. However, as observed in the case of the imported layer flock described above, such strains could easily be introduced into Slovenia by the poultry trade, especially by flocks that are reared and vaccinated in foreign countries and then imported into Slovenia.

Three ILTV strains (1315/18, 1718/18, and 1560/19) detected in one hobby flock and two backyard flocks, were almost identical to each other and very similar to the sequences of field strains detected in Russia, China, and the United States, as well as to sequences of TCO vaccine strains, and they formed a group that is similar to Clade II as proposed by Menendez et al. [3]. The reference strains for this clade are TCO vaccine strains such as Ivax US vaccine strains, the virulent USDA reference strain, and virulent isolate 81,658 from the United States. These strains are genetically obviously different from the other four Slovenian strains (Figure 2a,b). Since the TCO-based vaccines are mainly used in North America, South America, China, some countries in Southeast Asia, the Middle East, and South Africa [11], the scenario of the introduction of TCO vaccine strains into Slovenia by the commercial poultry trade seems somehow unlikely. It could only be speculated that these strains were introduced through frequent trading and contact with other poultry species at shows, which is typical for hobby flocks, or interaction with free-living birds in backyard flocks.

Our study confirms that ILTV strains from two genetically different clusters were detected in poultry flocks in Slovenia. Since there are no vaccination programs in Slovenia, these strains easily co-circulate in commercial poultry flocks and backyard/hobby flocks, which could result in recombination events and the emergence of new strains as has been described before [32].

Coinfections or secondary infections in diseases caused by viral pathogens such as ILTV may provide a more serious condition and lead to increased mortality [33,34]. Most reports of ILT mixed infections detected by molecular methods or the isolation of pathogens are associated with backyard flocks, although they have also been confirmed in commercial poultry [16,17,33,34,35].

Concomitant infections with other respiratory pathogens were detected by PCR in all flocks, although backyard or hobby flocks were positive for more respiratory pathogens compared to commercial flocks (Table 3). In all submissions, coinfection with MS was confirmed. The results are not surprising due to the high incidence of MS in commercial and backyard poultry in Slovenia [36,37]. Coinfection with MG was confirmed in backyard flocks but not in commercial layers. The main reason might be that infection with MG in commercial breeder poultry flocks in the EU is under strict control [38], which is not the case for MS. Similar results have also been reported by other authors. In a study of ILT outbreaks in backyard flocks in California, coinfection with MS and MG was found in approximately 30% of confirmed ILT cases [17], whereas in the US state of Maryland, mixed infection with ILT and MG was detected by PCR in one of 39 flocks [16]. In a prospective study of the prevalence of viral and bacterial pathogens in small poultry flocks in Ontario, Canada, ILT was detected in 16%, MS in 36%, and MG in 23% of chicken samples examined, and mixed infection with all three pathogens was confirmed in eight of 133 chicken submissions [35]. In a study of commercial layers infected with ILT, Couto et al. [34] found coinfection with *Mycoplasma* spp. in 22.6% of samples investigated.

Infection with IBV has been shown to increase susceptibility to secondary respiratory infections or increase the damage of infections with primary respiratory pathogens [35]. In addition, it was also shown that live attenuated vaccines such as that for IBV may promote respiratory infections [36]. In our study, IBV was detected in all backyard and commercial flocks tested. In Slovenia, vaccination against IBV is regularly performed in commercial poultry, but not in backyard/hobby flocks. Therefore, the IBV strains detected in these flocks could be wild-type IBV strains or vaccine strains that spilled over from vaccinated poultry flocks, as has been described previously [39]. Unfortunately, no further studies such as genotyping, isolation, or virulence determination have been performed that would allow differentiation between detected IBV strains. Most of the commercial layer flocks in Slovenia are vaccinated against IBV with live attenuated vaccines in the rearing period; only in regions with high field pressure of IBV infection are revaccinations with live attenuated vaccines also performed during production. This approach was used in the flock that had been imported from abroad and was vaccinated against ILT with the CEO vaccine (case ID1477/19). Severe respiratory disorders with a drop in egg production and increased daily mortality were observed 2 days after revaccination with live attenuated vaccine against IBV. It seems that vaccination against IBV acted as a trigger and reactivated the ILT vaccine virus [7,8]. Due to intense vaccination programs and biosecurity measures infections of commercial layer flocks with IBV are not common in Slovenia [40,41].

ORT was detected in three submissions. Owners reported severe respiratory signs and mortality (Table 1), and fibrinous pneumonia was found at necropsy (Table 2). Most experimental studies showed that inoculation only with ORT causes minimal pathological lesions, but in field conditions, infection with ORT can cause high mortality with gross lesions, including pneumonia, pleuritis, and airsacculitis, especially if there are concurrent infections with respiratory viruses or bacteria present [42].

Simultaneously occurring infections due to both ILT and APV are rarely described in the literature [34,43,44,45]. We confirmed coinfection with APV in only one submission of hens from a backyard flock, with severe fibrinous oculonasal discharge and diphtheric lesions spread over the entire length of the trachea, which are indicative for ILT and for the diphtheric form of APV.

Our study is the first to document ILT infection in Slovenia. Mixed respiratory infections are difficult to diagnose because usually only the identification of one pathogen constitutes a diagnosis, but as our study indicated, they could be expected under field conditions. Control of coinfections is an important factor in ILT management and should not be ignored [17]. In addition, the potential for pathogen exposure and spread from small flocks to commercial flocks should not be overlooked.

## Figures and Tables

**Table 1:** Description of poultry flocks with suspicion of ILT infection.

Case ID/ Year of Collection	Flock Description	Age	Clinical Signs
686/17	Backyard flock, 25 hens, different ages	Not known	Severe respiratory disorders, purulent conjunctivitis, diarrhea
1482/17	Backyard flock, 80 broilers and 45 hens	4 weeks, <1 year	Severe respiratory disorders and mortality seen in broilers, no clinical signs in adult hens
1104/18	Commercial layers, 6000 hens, farm with outdoor access ^1^	50 weeks	Mild respiratory disorders, drop in egg production (3%), higher daily mortality (0.81% per day)
1315/18	Backyard flock, 400 hens, two ages	>1 year, 20 weeks	Severe respiratory disorders and conjunctivitis observed only in younger hens
1718/18	Hobby flock, 420 hens, different ages	Not known	Nasal discharge, mild to severe respiratory disorders seen in 35% of birds
1477/19	Commercial layers, kept on multi-age farm, 6300 hens ^1,2^	25 weeks	Respiratory disorders, drop in egg production (c. 5%), higher daily mortality (0.24% per day)
1560/19	Commercial layers, 14,000 hens, multi-age farm ^1^	39 weeks	Respiratory disorders, slight drop in egg production (4.0%), higher daily mortality (0.12% per day)

^1^ During the rearing period, the birds were vaccinated against Marek disease, *Salmonella* Enteritidis, coccidiosis, infectious bursal disease, infectious bronchitis, Newcastle disease, fowl pox, and egg drop syndrome; ^2^ During the rearing period, the flock was vaccinated against infectious laryngotracheitis with live attenuated CEO vaccine Nobilis ILT**^®^**, and during the laying period the hens were vaccinated against infectious bronchitis virus with live attenuated vaccine.

**Table 2:** Primers sequences used for molecular detection of respiratory pathogens.

Pathogen	Primer/Probe	Sequence (5′-3′)	Reference
AIV	M + 25	AGATGAGTCTTCTAACCGAGGTCG	[18]
	M − 124	TGCAAAAACATCTTCAAGTCTCTG	
	M + 64	FAM-TCAGGCCCCCTCAAAGCCGA-TAMRA	
NCD (APMV1)	M + F4100	AGTGATGTGCTCGGACCTTC	[19]
	M − R4220	CCTGAGGAGAGGCATTTGCTA	
	M + 4169	FAM-TTCTCTAGCAGTGGGACAGCCTGC-TAMRA	
Coronaviruses (IBV)	11-FW	TGATGATGSNGTTGTNTGYTAYAA	[20]
	13-RV	GCATWGTRTGYTGNGARCARAATTC	
	probe III	FAM-TCTAARTGTTGGGTDGA-EDQ	
Herpesvirus	DFA	GAYTTYGCNAGYYTNTAYCC	[21]
	KG1	TCCTGGACAAGCAGCARNYSGCNMTNAA	
	ILK	GTCTTGCTCACCAGNTCNACNCCYTT	
	TGV	TGTAACTCGGTGTAYGGNTTYACNGGNGT	
	IYG	CACAGAGTCCGTRTCNCCRTADAT	
APV	HP444F	CAGCAGGTGCTAAACAACAA	[22]
	HP444R	CGGTAGCTTAACGCCGAATA	
MG	F	TTGGGTTTAGGGATTGGGATT	[23]
	R	CCAAGGGATTCAACCATCTT	
	TaqMan probe	FAM-TGATGATCCAAGAACGTGAAGAACACC-BHQ2	
MS	F	CTAAATACAATAGCCCAAGGCAA	
	R	CCTCCTTTCTTACGGAGTACA	
	TaqMan probe	FAM-AGCGATACACAACCGCTTTTAGAAT-BHQ1	
ORT	Ort 2F	GTCGCCGTAGTCATTAACCTCGTA	[24]
	Ort 2R	CTGTAGCCGATCAGCGTTTGAATG	
ILT	ICP4-1F	ACTGATAGCTTTTCGTACAGCACG	[15]
	ICP4-1R	CATCGGGACATTCTCCAGGTAGCA	
	ICP4-2F	CTTCAGACTCCAGCTCATCTG	
	ICP4-2R	AGTCATGCGTCTATGGCGTTGAC	

**Table 3:** Main pathological findings and detection of pathogens.

Case ID	No Examined Birds	Main Pathological Findings ^1^	Results of PCR Testing ^2^					
			ILTV	IBV	APV	MG	MS	ORT
686/17	2	Edema of the conjunctivae and severe fibrinous conjunctivitis, sinusitis, tracheitis and pneumonia, fibrinous oophoritis, infestation with *Ascaridia galli*, *Capillaria* sp., and *Dermanyssus gallinae* (2/2)	+	+	+	+	+	+
1482/17	2	Hyperemia of the conjunctivae, serofibrinous sinusitis, hollow caseous cast in larynx and upper trachea, fibrinous to caseous tracheitis, fibrinous pneumonia (2/2)	+	+	nd	+	+	+
1104/18	6	Mild conjunctivitis (6/6), fibrinous oophoritis and peritonitis (egg peritonitis) (3/6), infestation with *Ascaridia galli* (6/6)	+	+	nd	+	+	−
1315/18	5	Conjunctivitis (5/5), serofibrinous sinusitis (5/5), fibrinous to caseous tracheitis (3/5), serofibrinous airsacculitis, peritonitis and oophoritis (3/5), infestation with *Ascaridia galli* and *Capillaria* sp. (2/5)	+	nd	−	−	+	nd
1718/18	1	Severe fibrinous conjunctivitis, yellow caseous exudate in beak cavity, hemorrhagic to caseous laryngitis and tracheitis, serofibrinous sinusitis (1/1)	+	nd	nd	+	+	+
1477/19	4	Hyperemia of conjunctivae (4/4), serofibrinous sinusitis (4/5), hollow caseous cast in larynx and upper trachea (3/4), fibrinous to caseous tracheitis (3/4)	+	+	nd	−	+	nd
1560/19	6	Conjunctivitis (5/6), serofibrinous sinusitis (5/6), fibrinous to caseous tracheitis (4/6), serofibrinous airsacculitis (5/6), fibrinous peritonitis and oophoritis (4/6), infestation with *Ascaridia galli* (3/6)	+	+	nd	−	+	nd

^1^ The number of birds with a specific pathologic lesion/number of all birds examined from each submission is given in parentheses. ^2^ Molecular results are presented as: nd = not done, + positive, − negative.

## Data Availability

Not applicable.
